# Selection of Reference Genes for Quantitative Real Time PCR (qPCR) Assays in Tissue from Human Ascending Aorta

**DOI:** 10.1371/journal.pone.0097449

**Published:** 2014-05-19

**Authors:** Carmen Rueda-Martínez, Oscar Lamas, María José Mataró, Juan Robledo-Carmona, Gemma Sánchez-Espín, Manuel Jiménez-Navarro, Miguel Such-Martínez, Borja Fernández

**Affiliations:** 1 UGC del Corazón, Instituto de Investigación Biomédica de Málaga (IBIMA), Hospital Clínico Universitario Virgen de la Victoria, Málaga, Spain; 2 Departamento de Medicina, Facultad de Medicina, Universidad de Málaga, Málaga, Spain; 3 Departamento de Biología Animal, Instituto de Investigación Biomédica de Málaga (IBIMA), Facultad de Ciencias, Universidad de Málaga, Málaga, Spain; University of Jaén, Spain

## Abstract

Dilatation of the ascending aorta (AAD) is a prevalent aortopathy that occurs frequently associated with bicuspid aortic valve (BAV), the most common human congenital cardiac malformation. The molecular mechanisms leading to AAD associated with BAV are still poorly understood. The search for differentially expressed genes in diseased tissue by quantitative real-time PCR (qPCR) is an invaluable tool to fill this gap. However, studies dedicated to identify reference genes necessary for normalization of mRNA expression in aortic tissue are scarce. In this report, we evaluate the qPCR expression of six candidate reference genes in tissue from the ascending aorta of 52 patients with a variety of clinical and demographic characteristics, normal and dilated aortas, and different morphologies of the aortic valve (normal aorta and normal valve n = 30; dilated aorta and normal valve n = 10; normal aorta and BAV n = 4; dilated aorta and BAV n = 8). The expression stability of the candidate reference genes was determined with three statistical algorithms, GeNorm, NormFinder and Bestkeeper. The expression analyses showed that the most stable genes for the three algorithms employed were CDKN1β, POLR2A and CASC3, independently of the structure of the aorta and the valve morphology. In conclusion, we propose the use of these three genes as reference genes for mRNA expression analysis in human ascending aorta. However, we suggest searching for specific reference genes when conducting qPCR experiments with new cohort of samples.

## Introduction

Ascending aortic dilatation (AAD) is a prevalent human aortopathy that may lead to dissection and rupture of the artery with fatal consequences [1], [2]. AAD occurs frequently in association with bicuspid aortic valve (BAV), which constitutes the most frequent congenital cardiac malformation, with an incidence in the general population between 0.5% and 2% [2]–[4]. Although hemodynamic stress on the aortic wall caused by the abnormal valve anatomy has been historically adduced as the causal factor of the association between BAV and AAD, the most accepted current hypothesis proposes that patients with BAV present structural defects of the aortic media that predispose to the aortopathy [2], [4]–[6].

The pathogenetic mechanisms leading to AAD are still poorly understood, particularly in patients with BAV. The alteration of the expression of Fibrillin-1 and Metalloproteases in the aortic wall of BAV patients suggests that matrix homeostasis is a key biological process for the disease progression [2], [4], [6]. However, contradictory results from independent studies [7], [8], and absence of further significant evidences from associated genetic pathways have slowed the progress in this field. It seems clear that systematic research on gene expression in aortas of patients with AAD and BAV is required.

Nowadays, the most useful tool to quantify gene expression is the quantitative real-time PCR (RT-qPCR) technique [9]. Normalization methods are used to ensure accurate and suitable quantification of PCR data. These methods enable to control the variation in the extraction process, the reverse transcription yield, and other factors contributing to experimental variability, thus allowing the comparison of mRNA concentration across different samples [10]. The use of reference genes as an internal control is the most common method employed for mRNA expression normalization [11]. Glyceraldehyde-3-phosphate dehydrogenase (GAPDH), β-Actin (ACTB), α-Actin (ACTA) and ribosomal RNA (18S) are the most commonly chosen reference genes for normalization in qPCR analyses. However, they are frequently used without further validation. In several reports, these classical reference genes revealed different expression levels, and were evaluated as inadequate for analyzing gene expression in cardiac and extra cardiac tissues [11]–[19]. Moreover, in a recent paper [19], the expression of 32 possible reference genes was evaluated in aortic tissue of patients with normal and diseased arteries by the RT-qPCR method. The study revealed relatively low expression stability of classical reference genes, indicating their reduced suitability for gene expression studies involving aortic tissue.

In this context, we decided to explore the expression stability of the genes ABL1, HMBS, CASC3, CDKN1β, POLR2A and TBP, to test whether one or more of these genes can be used as reference genes for aortic wall tissue gene expression, regardless of aortic structural abnormalities and valve morphology. These genes have been previously used as reference genes in studies involving human and animal heart tissue [17], [20]–[22]. To determinate the stability of candidate reference genes, we took advantage of three statistical algorithms GeNorm, NormFinder and Bestkeeper, which are commonly used methods to identify the most stable reference genes for qPCR data normalization [23]–[25].

## Materials and Methods

### Patient sample

Tissue samples from the ascending aorta were collected from 52 patients subjected to cardiovascular surgery at the Virgen de la Victoria Hospital. Experimental procedures were approved by Ethics and Research Committee of Malaga accordingly to the Committee's guidelines for Human Tissue Research (Malaga, Spain) and to the declaration of Helsinki with written informed consent obtained from all patients involved in the study.

The valve morphology (bicuspid or tricuspid) and the size of the ascending aorta were evaluated by image echocardiography according to Robledo-Carmona et al. [26]. Demographic and clinical parameters of the patients are detailed in table 1.

**Table 1:** Demographic and clinical characteristics of patients.

	NDTAV	DTAV	NDBAV	DBAV
**Demographic parameters**				
N (52)	30	10	4	8
Age (years) (mean±SD)	67,7±9,57*	64±8,15	50±8,73	60,5±11,22
Sex (male/female)	24/6	6/4	2/2	6/2
BMI (mean±SD)	27,9±3,66	29±4,73	25,4±2,52	25,9±2,84
**Clinical parameters**				
Smoking	9 (30)	4 (40)	4 (100)	5 (62,5)
Hypertension	19 (63.3)	7(70)	2(50)	3 (37,5)
Diabetes mellitus	13 (43,3)	1(10)	0 (0)	3(37,5)
Obesity	4 (13,3)	5 (50)	0 (0)	1 (12,5)
Dyslipemia	14 (46,6)	4 (40)	0 (0)	3(37,5)
EF (%±SD)	57,8±12,27	58.6±10,48	53±17,04	54,8±17,28

BMI: Body mass index; DBAV: Dilated Bicuspid Aortic Valve; DTAV: Dilated Tricuspid Aortic Valve; EF: Ejection fraction; NDBAV: Non Dilated Bicuspid Aortic Valve; NDTAV: Non Dilated Tricuspid Aortic Valve. Values of clinical parameters are shown as total number of patients with percentage between parenthesis.

*: significantly different to NDBAV (p<0.05).

The samples were divided in 4 groups depending on the valve morphology and the structural features of the aortic wall: patients with normal, tricuspid aortic valve (TAV) and normal size aorta (NDTAV); patients with TAV and dilated aorta (DTAV); patients with BAV and normal aorta (NDBAV); patients with BAV and dilated aorta (DBAV) (Fig. S1). Comparisons of qualitative and quantitative demographic and clinical characteristic between the 4 groups of patients were performed using the χ^2^ test and the non parametric Kruskal-Wallis test, respectively. The SSPS software version 15 was used for the statistic analyses.

### Tissue collection and RNA isolation

Tissue samples from the ascending aorta were collected at the operation room, immediately immersed in liquid nitrogen, and further stored at −80°C for subsequent RNA extraction. Each sample was homogenized using IKA ultra-turrax T10B basic homogenizer (LABOTAQ, Spain). Total RNA (Table S1) was extracted using the mirVana Paris Kit (Ambion, Germany) following the manufacturer's instructions, and treated with DNasa I (Quiagen, Germany) in order to eliminate any trace of genomic DNA. RNA concentration and purity were evaluated with a Nanodrop D-1000 spectrophotometer (Nanodrop technologies, Wilmintogton, DE, USA). Only samples with an OD260/280 ratio from 1.8 to 2.1 were selected for determination of mRNA expression by qPCR. In addition, RNA integrity was confirmed by 1.2% denaturing-formaldehyde agarose gel electrophoresis (Fig. S2).

### cDNA Synthesis

RNA samples were reverse transcribed to cDNA using the High Capacity cDNA Reverse Transcription Kit (Applied Biosystems, CA, USA) according to the manufacturer's recommendations. The reverse transcriptase reaction was performed with 100 ng of RNA in a final volume of 20 µl (Table S1). The thermocycler conditions consisted of a first step at 25°C for 10 min, followed by a second step at 37°C for 120 min. The samples were then heated at 85°C for 5 min and cooled to 4°C. The cDNA was stored at −20°C until further analysis.

### Real time quantitative PCR (qPCR)

The cDNA was analyzed by qPCR using the TaqMan Gene Expression Master Mix (Applied Biosystems, CA, USA) in an ABI PRISM7500 FastReal-Time PCR Instrument (Applied Biosystems, CA, USA) following the manufacturer's instructions. An amount of 10 ng of cDNA was used for each reaction and the total reaction volume was 20 µl per well. Each pair of primers was tested in independent plates together with all the samples and a negative control (No Template Control) was included on each plate in order to check general contamination. Direct detection of PCR products was monitored by measuring the increase in fluorescence of the FAM dye with an internal passive reference ROX dye. The cycling conditions were two holding stages, the first at 50°C for 20 sec and the second at 95°C for 10 min, followed by 40 cycles at 95°C for 15 sec, and at 60°C for 1 min. We studied the expression of 6 reference genes: ABL1, HMBS, CASC3, CDKN1β, POLR2A, and TBP. These candidate genes have been previously used as reference genes in comparative expression studies of different tissues [18], [27], including the cardiovascular system [17], [20]–[22]. In the ABI 7500 software, the threshold was manually adjusted at the beginning of the exponential phase of amplification. Baseline was automatically calculated by the software. The delta-Ct method was used for quantification of Ct values. The information about the specific Taqman assays (primers + hydrolysis probe) used for amplification is shown in table 2 (Applied Biosystems, CA, USA). The primers were designed to span an exon/exon junction.

**Table 2:** Candidate reference genes.

Symbol	Function	Assays ID
HMBS	Heme synthesis, porphyrin metabolism	Hs00609297_ml
ABL1	Protooncogene, tyrosine kinase	Hs00245445_ml
POLR2A	DNA-directed RNA polymerase; transcription	Hs00172187_ml
CDKN1β	Cell cycle inhibitor	Hs00153277_ml
CASC3	Linked to development of breast cancer	Hs00201226_ml
TBP	Transcription factor	Hs99999910_ml

Usually, PCR efficiencies are calculated using the dilution or standard curve method. With this method, PCR efficiencies may vary depending on the input concentration [28]. Several studies have shown a large variation in efficiency values when standard curves are replicated [29]–[31]. To solve this issue, we employed the LinRegPCR version 2012.x software to calculate the accurate amplification efficiency (E) and correlation coefficient (R^2^) of each pair of primers [32]–[34]. This software determines PCR efficiency from the slopes of the exponential phase of the individual amplification curves (E = 10^Slope^) and calculates the amplification mean of each amplicon. The correlation coefficient is indicative of the noise in the subset of data points used in the exponential phase to determinate the PCR amplification efficiency [28], [34].

### Expression stability of candidate reference genes

The stability of the candidate reference genes was evaluated by three software algorithms: GeNorm [23], [35], NormFinder [24], [36] and Bestkeeper [25], [37]. The Ct values were transformed into relative quantity data for GeNorm and NormFinder algorithms, using the delta-Ct method: Q = E^ΔCt^ where E =  amplification efficiency of each amplicon, and ΔCt =  lowest Ct value - sample Ct value. For Bestkeeper, the raw values of amplification efficiency and Ct were introduced into the software.

### GeNorm analysis

The GeNorm algorithm is used to identify reference genes with the most stable expression in different tissues or treatment conditions [23], [35]. The software defines two parameters to quantify stability: the expression stability (M value) and the pair-wise variation (V value). The gene with the lowest M value is considered to have the most stable expression. V values were proposed by Vandesompele J et al. [35] as a guide to determine the optimal number of candidate reference genes required for normalization, defining it as a 0.15 cut-off value. If V value is below or equal to 0.15, it is not necessary to include additional reference genes for normalization.

### NormFinder analysis

The NormFinder program is a Visual Basic application tool for Microsoft Excel used to select reference genes among a set of candidates genes for optimal normalization [24], [36]. As in the GeNorm method, the gene with the lowest stability value (M) is the most stable expressed gene. NormFinder takes into account intragroup and intergroup variations in stability, ranking the best two reference genes for normalization.

### Bestkeeper analysis

The Bestkeeper software is also a tool to determine the most stable reference genes based on the analysis of the correlation coefficient of all possible pairs of candidate reference genes [25], [37]. The software determines the Bestkeeper index (BI), which is the geometric mean of the Ct values of the highly correlated candidate reference genes [37]. The reference genes are identified as the most stable genes when they exhibit the lowest standard deviation (SD) and highest correlation coefficient (r). Genes that show a SD greater than 1 are considered unacceptable.

## Results

### Patients Features

The demographic and clinical characteristics of the patients are shown in table 1. No statistical significant differences were found among the four groups studied, except for the age between the groups NDTAV and NDBAV (p = 0,032).

### Expression profiles of candidate reference genes

**Figure 1:**
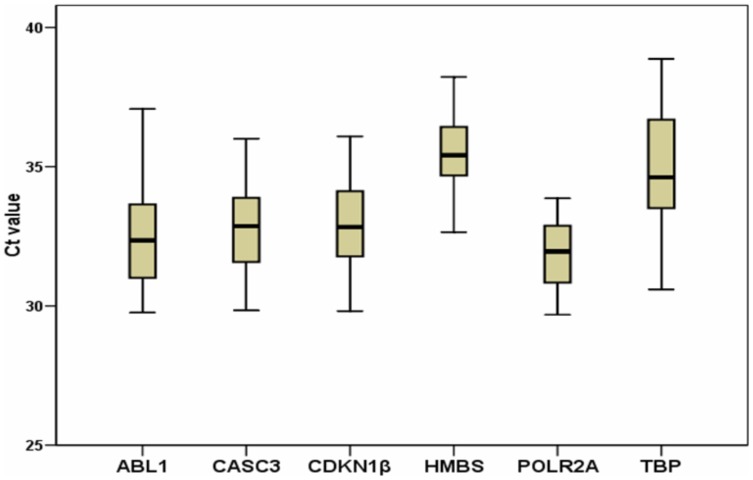
Expression levels of the six candidate reference genes.

The expression levels of the six candidate reference genes were determined in the 52 human samples of ascending aorta. A single band of the expected size for each amplicon was observed in a 2% agarose gel, and no bands were detected in the no template controls (NTCs), that was included for each specific pair of primers in each plate. The NTC fluorescence levels were below the detection limit in all cases (Table S1). The raw Ct or Cq expression values (Table S1) were used to calculate the mean Ct for each amplicon in the samples (Fig. 1). The candidate reference genes showed mean Ct values between 32 and 35.

Amplification efficiencies and correlation coefficients were analyzed for the 6 candidate reference genes using the LinRegPCR software. The amplicon length, the mean amplification efficiencies and the correlation coefficient of each amplicon are detailed in table 3. Efficiencies ranged between 1.8 (90%) and 2 (100%), indicating a correct amplification of all amplicons. The correlation coefficient ranged between 0.963 and 1, indicating a high linearity of all curves.

**Table 3:** Amplicon length (A), correlation coefficient (R^2^), and amplification efficiencies (E%) of reference genes.

Symbol	Name Gene	A	R^2^	E
HMBS	hydroxymethylbilane synthase	64	0,963	95
ABL1	non-receptor tyrosine kinase	91	0,999	90
POLR2A	polymerase (RNA) II (DNA directed) polypeptide A	61	0,999	100
CDKN1β	cyclin-dependent kinase inhibitor 1B	71	1	95
CASC3	cancer susceptibility candidate 3	67	1	95
TBP	TATA box binding protein	127	0,993	95

### GeNorm analysis

**Figure 2:**
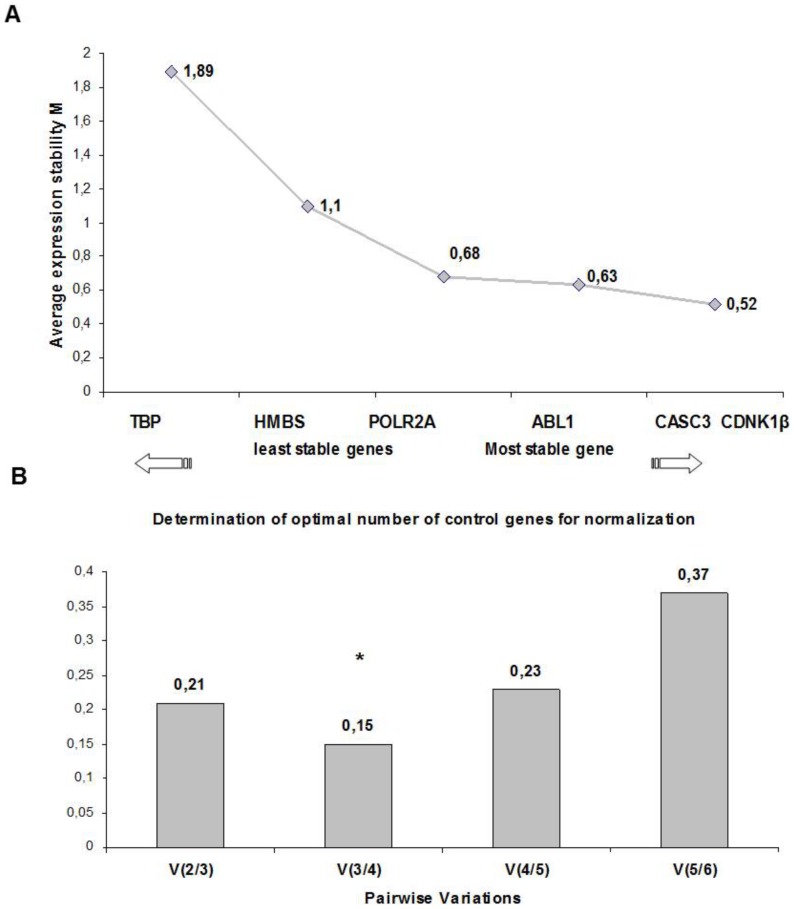
Stability values (A) and pair-wise variation values (B) of reference genes obtained by GeNorm. A pair wise variation value of 0.15 was obtained for V_3/4_ (B), indicating that three genes are required for normalization according to GeNorm. The three most stable reference genes suggested by GeNorm were CDKN1β, CASC3, and ABL1 (A). Note that POLR2A also shows a low M stability value (0.68), similar to that of ABL1 (0.63).

The candidate reference genes showed M values (stability values) below 1.5 except for TBP with an M value of 1.89 (Fig. 2A). Therefore the gene TBP was considered unsuitable by GeNorm software. The gene HMBS also showed a high M stability value (1.10). The genes POLR2A and ABL1 showed similar and intermediate M values (0.68 and 0.63 respectively), whereas CDKN1β and CASC3 showed the lowest M value (0.52), thus representing the most stable reference genes (Fig. 2A). A pair-wise variation (V) value of 0.15 was obtained for V_3/4_ (Fig. 2B), indicating that three genes with the lowest M values are required for accurate normalization. Thus, GeNorm analysis suggested that CDKN1β, CASC3 and ABL1 are the most suitable genes to normalize mRNA expression for our samples.

### NormFinder Analysis

**Figure 3:**
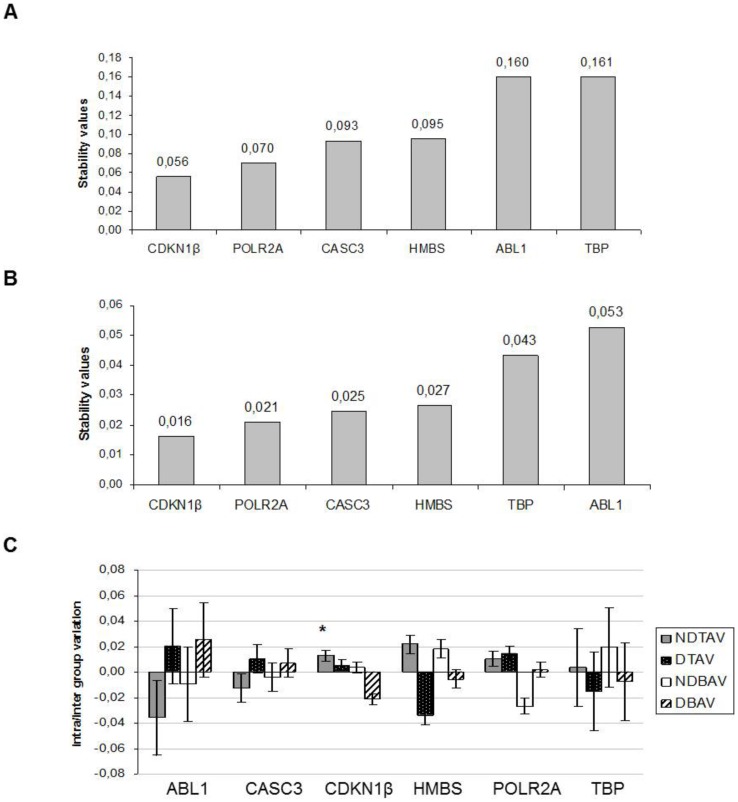
Stability values of reference genes and their variation obtained by NormFinder. A) Stability values considering all the samples in one group. B) Stability values considering the four groups of samples. C) Variation of the stability values in the four groups of samples. In C, the columns represent the inter-group variation and the error bars represent the intra-group variation of the stability value of each reference gene.

In the NormFinder analysis, the most stable reference gene (lowest M value) for the samples studied was the CDKN1β gene (0.056), followed by POLR2A (0.070) and CASC3 (0.093) (Fig. 3A). When considering the four groups of patients (NDTAV, DTAV, NDBAV and DBAV), the same three genes had the lowest stability values: CDKN1β (0.016), POLR2A (0.021), and CASC3 (0.025) (Fig. 3B). These three genes showed also the best combination of intra and inter group variation (Fig. 3C), with a combined stability value for CDKN1β and POLR2A of 0.014 (data not shown).

### Bestkeeper analysis

The genes ABL1 and TBP exhibited a SD value above 1, indicating that they are the candidate reference genes with less stability, below the level of acceptance of the test (Table 4). POLR2A and CDKN1β were the most stable reference genes with a combination of the lowest SD, and the highest coefficients of correlation. CASC3 also showed a quite high coefficient of correlation, but the combination of this coefficient with SD values was inferior to that of POLR2A and CDKN1β.

**Table 4:** Description statistic of the candidate reference genes based on Bestkeeper analysis.

Genes	POLR2A	CDKN1β	CASC3	HMBS	ABL1	TBP	BI
Geo mean	32.02	32.86	32.83	35.51	32.33	34.77	33.3
Min	29.68	32.65	29.83	32.65	29.73	30.59	30.9
Max	34.41	38.96	36.35	38.96	35.81	38.87	35.9
Std dev	0.91	0.99	1	1	1.2	1.6	0.99
Std dev (x-fold)	1.70	1.79	1.89	1.81	2.02	2.56	1.92
Coeff of corr. (r)	0.92	0.97	0.94	0.85	0.86	0.80	
p-value	0.001	0.001	0.001	0.001	0.001	0.001	

B.I.: Bestkeeper Index; Geo mean: geometric mean of Cp values; Min, Max: minimum and maximum values of Cp.

In summary, the Bestkeeper analysis indicated that both POLR2A and CDKN1β, followed by CASC3 are the most stable genes in agreement with NormFinder analysis.

## Discussion

During the last decade, numerous studies have employed qPCR to find differentially expressed genes in the aneurysmatic aorta of patients with BAV [38]–[48]. These studies are important because we still do not have relevant biomarkers of BAV-associated aortopathy, and because the aetio-pathological mechanisms leading to AAD in patients with BAV are poorly understood. In man, this pathological entity is more frequent than AAD associated with other connective tissue disorders like Marfan syndrome [46]. In the studies cited above, a variety of reference genes were used for normalization, including GAPDH [38]–[41], 18S [42], [43], TBP [44], SN0202 [45], RNU6 [45], RPLP0 [47], [48], or total RNA [49]. However, in none of these studies the reference genes employed for normalization were validated by any available method.

Several studies have reported that some of the classical reference genes employed in gene expression experiments (GAPDH, ACTA, ACTB, 18S, and 28S) are not adequate to normalize gene expression in human myocardium [12], cardiac valve tissue [14] and aortic tissue [19], probably because their expression can be affected by drugs and other factors [15], [16]. This is particularly relevant in research on the cardiovascular system, where different treatments and clinical conditions may affect basal gene expression levels. In this study, we analyze the expression patterns of several genes in the wall of human ascending aortas, in order to assess their efficiency as reference genes in studies of gene expression quantification.

The samples studied belonged to four groups of patients with and without AAD, different valve morphologies (TAV and BAV), and with a variety of clinical and demographic characteristics. To select the most efficient reference genes we employed three different algorithms GeNorm, NormFinder, and Bestkeeper. These are currently considered the gold standard methods for the selection of appropriate reference genes for normalization in gene expression experiments involving RT-qPCR [12], [18], [20], [21], [27], [28], [35]–[37], [50]. The candidate genes tested for selection of the best reference genes were ABL1, CASC3, CDKN1β, POLR2A, HMBS, and TBP. All these genes have been previously selected as the most stable genes in cardiovascular studies involving human and rodent myocardial tissue [17], [20]–[22].

The candidate reference genes showed raw Ct values ranging from 32 to 35. In addition, a high amplification efficiency of each candidate gene was obtained with the LinRegPCR. The efficiency values were 95% and 100%, except for ABL1 (90%) (Table 3). In addition, the correlation coefficient of the amplicons showed a very high linearity, with values higher than 0.99 except for HMBS (0.963) (Table 3). These data indicate that despite of very low mRNA expression levels, the efficiencies of amplification and the correlation coefficients of all the candidate reference genes were acceptable for subsequent analysis of stability.

The results of the GeNorm analysis revealed that the most stable reference genes for the samples studied were CDKN1β and CASC3, followed by ABL1, which showed the lowest M values (0.52 and 0.63, respectively) (Fig. 2A). In addition, the pair-wise variation analysis used by GeNorm suggested that the three most stable genes should be used for normalization (Fig. 2B). In contrast to GeNorm, NormFinder algorithm corrects intra and inter-group variation when studying a heterogeneous population [36]. When the stability of expression was calculated taking into account our four groups of patients (NDTAV, DTAV, NDBAV, and DBAV), the three most stable genes proposed by NormFinder were CDKN1β, POLR2A, and CASC3 (Fig. 3B). Furthermore, NormFinder indicated that the stability values of these genes showed the lowest intra/inter group variations for human ascending aorta tissue (Fig. 3C). While GeNorm and NormFinder softwares transform the raw Ct into relative values, the Bestkeeper algorithm uses the raw Ct values to analyze the stability of candidate reference genes [35]–[37]. Bestkeeper algorithm identified POLR2A and CDKN1β as the most stable genes for normalization, with the best combination of coefficient of correlation and SD, followed by CASC3 (Tables 4 and 5).

**Table 5:** Ranking of reference genes according to GeNorm, NormFinder and Bestkeeper algorithms.

Ranking	GeNorm	NormFinder	Bestkeeper
1	CDKN1β/CASC3	CDKN1β	POLR2A
2	ABL1	POLR2A	CDKN1β
3	POLR2A	CASC3	CASC3
4	HMS	HMBS	HMBS
5	TBP	ABL1	ABL1
6		TBP	TBP

The three algorithms employed resulted in slightly different rankings of genes in term of stability (Table 5). This variation is most probably due to differences in input data, parameters, and mathematical models employed by the software, as a similar variety of results have been published elsewhere [50], [51]. However, our results showed that two of the genes analyzed, CDKN1β and CASC3, were among the three most stable genes for the three algorithms employed in this study (Table 5). In addition, POLR2A was the first and second most stable gene for two of the three algorithms (Bestkeeper and NormFinder respectively), and the third for GeNorm, with a relatively low M value (Fig. 2A). These results indicate, with a high level of consistency, that CDKN1β, CASC3, and POLR2A are the most stable reference genes for human ascending aortic tissue in our patient population.

In a recent study, Henn et al. [19] identified EIF2B1, ELF1 and PPIA as the best reference genes for RT-qPCR analyses of human ascending aortic tissue. These reference genes were selected among 32 candidates, including the 6 candidate genes tested by us in the present study that were ranked between positions 7 and 25. Henn et al. employed the GeNorm algorithm alone to determine the most stable reference gene, followed by a hierarchic statistical design to differentiate the effects of valve morphology and aortic defect [19]. In our design, these effects are determined by the inter-group variation analysis performed by the NormFinder algorithm. In our opinion, this design is more efficient and simple, because NormFinder differentiates the effects of both inter- and intra-group variation of gene stability, without the need of an additional and external statistical approach. In addition, in our study we combine three different algorithms, GeNorm, NormFinder, and Bestkeeper to select the most stable reference genes in our four groups of patients. Thus, the fact that three independent algorithms agree in ranking the most stable reference genes adds a level of consistency to the results. In conclusion, even when applying specific methods for the selection of appropriate reference genes for normalization, the selected reference genes may vary among experiments. These variations may depend on the methodological strategy followed or on the patient population under study, what would imply that each cohort of patients to be compared requires a specific search for the most stable reference gene, when qPCR is to be used.

In summary, we propose the use of CDKN1β, POLR2A and CASC3, as reference genes for mRNA quantification in future studies that involve samples of ascending aorta from human patients with normal or dilated aortas and different morphologies of the aortic valve. However, when new cohorts of tissue samples are used, we suggest performing specific gene expression studies, in order to identify the most stable reference genes to be used for normalization.

## Supporting Information

Figure S1 **Representative echocardiographic images of a bicuspid aortic valve (A); a tricuspid aortic valve (C); a dilated tubular aorta (B); and a normal aorta (D).** A and C: short paraesternal views; B and D: long paraesternal views.
(PPT)

Figure S2 **Representative image of a denaturing agarose gel to check RNA integrity.** Two bright bands corresponding to ribosomal 28S rRNA and 18S rRNA are observed.
(PPT)

Table S1 **Weight of the tissue (Weight; mg), total RNA extracted (TRNA; ng), RNA input for reverse transcription (IRNA; ng), and Ct values of the reference genes for all the samples analyzed.**
(DOC)
