# Supplementary material for: Selection of Reference Genes for Quantitative Real Time PCR (qPCR) Assays in Tissue from Human Ascending Aorta
Source: PLoS One. 2014 May 19;9(5):e97449. doi: 10.1371/journal.pone.0097449 (PMC4026239; doi:10.1371/journal.pone.0097449)
Supplement: Figure S1 — Representative echocardiographic images of a bicuspid aortic valve (A); a tricuspid aortic valve (C); a dilated tubular aorta (B); and a normal aorta (D). A and C: short paraesternal views; B and D: long paraesternal views. (PPT) [file pone.0097449.s001.ppt]

## Slide 1
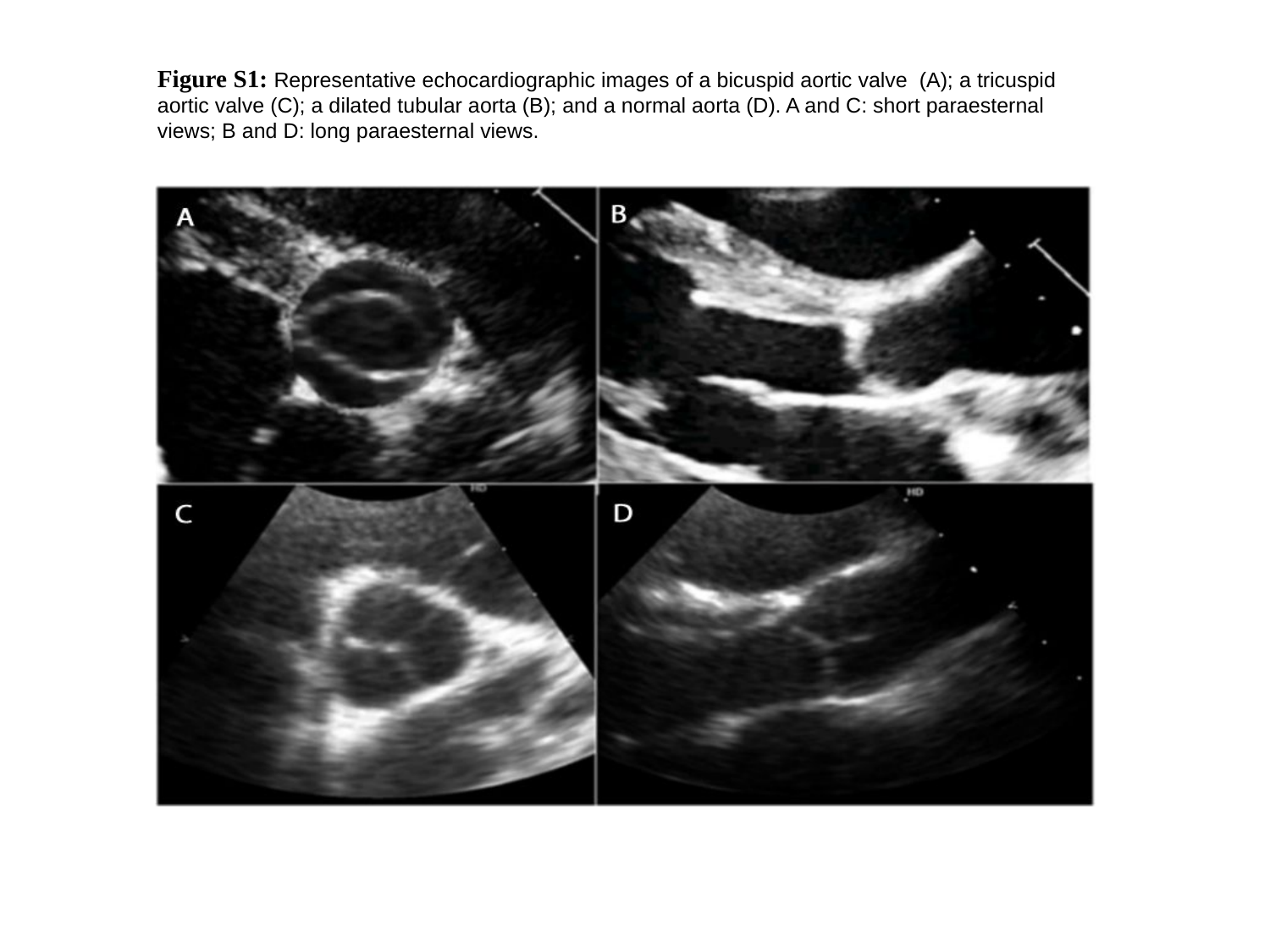

Figure S1: Representative echocardiographic images of a bicuspid aortic valve (A); a tricuspid aortic valve (C); a dilated tubular aorta (B); and a normal aorta (D). A and C: short paraesternal views; B and D: long paraesternal views.
