# Supplementary material for: Selection of Reference Genes for Quantitative Real Time PCR (qPCR) Assays in Tissue from Human Ascending Aorta
Source: PLoS One. 2014 May 19;9(5):e97449. doi: 10.1371/journal.pone.0097449 (PMC4026239; doi:10.1371/journal.pone.0097449)
Supplement: Figure S2 — Representative image of a denaturing agarose gel to check RNA integrity. Two bright bands corresponding to ribosomal 28S rRNA and 18S rRNA are observed. (PPT) [file pone.0097449.s002.ppt]

## Slide 1
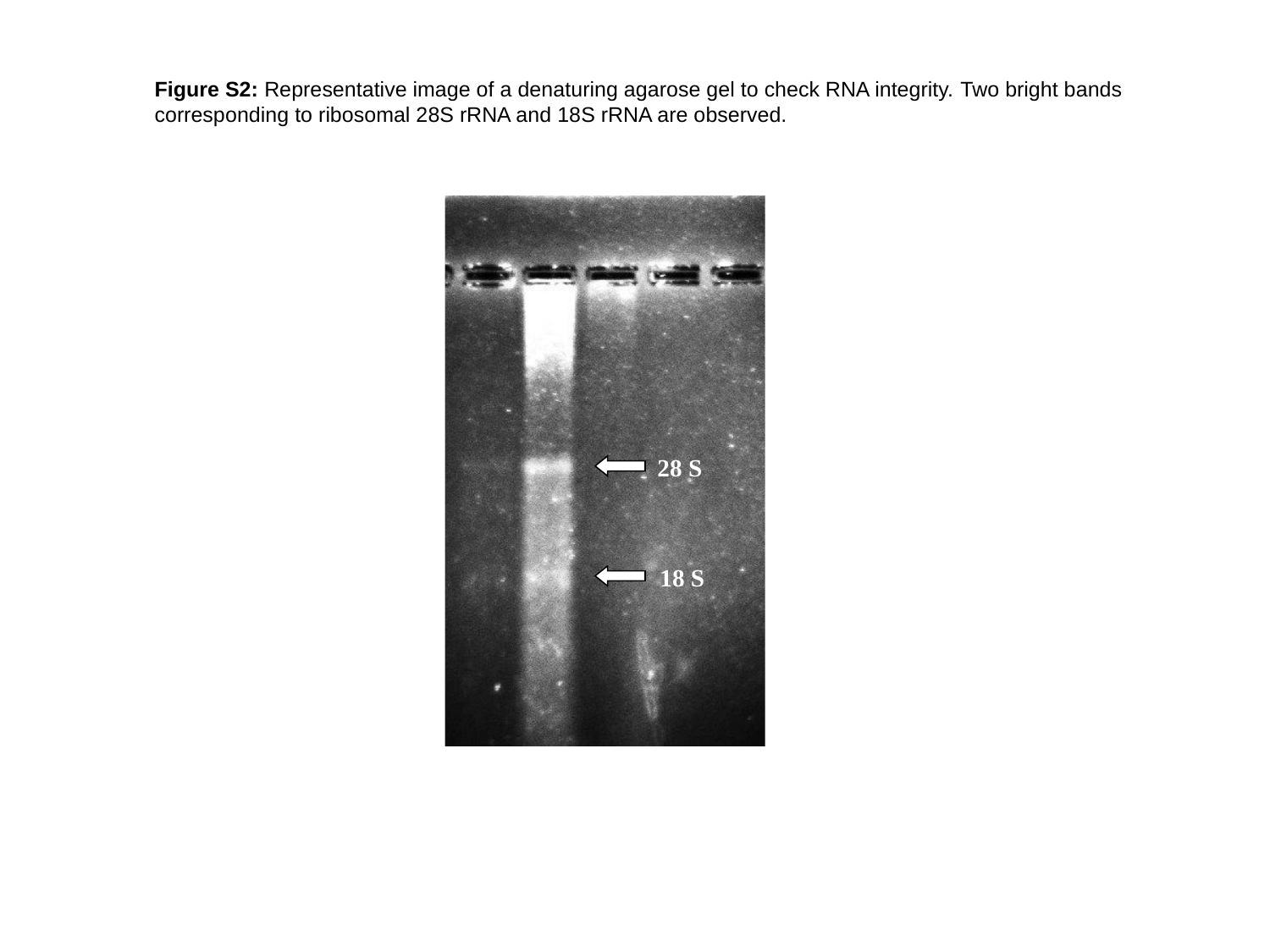

Figure S2: Representative image of a denaturing agarose gel to check RNA integrity. Two bright bands corresponding to ribosomal 28S rRNA and 18S rRNA are observed.
28 S
 18 S
