# Supplementary material for: Selection of Reference Genes for Quantitative Real Time PCR (qPCR) Assays in Tissue from Human Ascending Aorta
Source: PLoS One. 2014 May 19;9(5):e97449. doi: 10.1371/journal.pone.0097449 (PMC4026239; doi:10.1371/journal.pone.0097449)
Supplement: Table S1 — Weight of the tissue (Weight; mg), total RNA extracted (TRNA; ng), RNA input for reverse transcription (IRNA; ng), and Ct values of the reference genes for all the samples analyzed. (DOC) [file pone.0097449.s003.doc]

**Table Sup 1:** Weight of the tissue (Weight; mg), total RNA extracted (TRNA; ng), RNA input for reverse transcription (IRNA; ng), and Ct values of the reference genes for all the samples analyzed.

| **Samples** | **Weight** | **TRNA** | **IRNA** | **ABL1** | **CASC3** | **CDKN1ß** | **HMBS** | **POLR2A** | **TBP** |
| --- | --- | --- | --- | --- | --- | --- | --- | --- | --- |
| **1** | 12,3 | 640 | 100 | 32,61 | 33,01 | 33,09 | 34,55 | 32,25 | 35,34 |
| **2** | 17,8 | 795 | 100 | 34,98 | 34,96 | 34,52 | 37,94 | 33,48 | 36,73 |
| **3** | 11,8 | 560 | 100 | 32,67 | 33,58 | 33,67 | 34,50 | 32,49 | 36,17 |
| **4** | 4,5 | 390 | 100 | 33,27 | 32,97 | 33,45 | 35,99 | 32,71 | 34,21 |
| **5** | 12 | 580 | 100 | 37,15 | 37,10 | 36,03 | 37,26 | 33,65 | * |
| **6** | 7,6 | 725 | 100 | 35,75 | 36,02 | 34,96 | 37,88 | 34,08 | 36,66 |
| **7** | 12,6 | 610 | 100 | 33,38 | 33,90 | 33,98 | 34,81 | 33,58 | 36,98 |
| **8** | 4,5 | 390 | 100 | 33,87 | 33,80 | 33,96 | 35,20 | 32,82 | 37,62 |
| **9** | 9,4 | 510 | 100 | 32,67 | 32,56 | 32,15 | 34,40 | 31,41 | 32,22 |
| **10** | 11 | 675 | 100 | 34,25 | 35,29 | 35,00 | 36,63 | 33,31 | 37,60 |
| **11** | 9,5 | 435 | 100 | 31,13 | 31,36 | 31,65 | 35,01 | 30,79 | 33,75 |
| **12** | 5,7 | 405 | 100 | 30,43 | 30,50 | 30,93 | 32,65 | 30,44 | 31,75 |
| **13** | 14,7 | 550 | 100 | 33,74 | 33,58 | 33,32 | 37,48 | 32,23 | 33,91 |
| **14** | 13,7 | 685 | 100 | 34,13 | 34,60 | 34,03 | 36,88 | 32,75 | * |
| **15** | 6,7 | 415 | 100 | 33,29 | 33,54 | 33,98 | 36,41 | 32,58 | * |
| **16** | 16,4 | 850 | 100 | 30,89 | 30,37 | 30,36 | 33,75 | 30,59 | 31,20 |
| **17** | 4,6 | 385 | 100 | 35,01 | 36,35 | 35,38 | 36,97 | 34,41 | 35,96 |
| **18** | 30,7 | 2030 | 100 | 31,54 | 32,93 | 33,04 | 34,95 | 32,09 | 37,24 |
| **19** | 9,4 | 480 | 100 | 32,51 | 33,27 | 32,82 | 36,25 | 31,82 | 37,33 |
| **20** | 8,2 | 410 | 100 | 31,87 | 33,34 | 33,61 | 36,06 | 32,17 | * |
| **21** | 5,5 | 475 | 100 | 31,16 | 32,23 | 32,87 | 35,60 | 32,66 | 33,76 |
| **22** | 9,7 | 495 | 100 | 31,65 | 32,36 | 33,13 | 36,23 | 32,23 | 34,43 |
| **23** | 7,8 | 555 | 100 | 34,29 | 34,56 | 34,32 | 38,96 | 33,90 | * |
| **24** | 8,1 | 645 | 100 | 31,00 | 31,81 | 32,35 | 35,29 | 31,59 | 34,85 |
| **25** | 10,7 | 435 | 100 | 29,73 | 31,20 | 31,49 | 34,47 | 30,63 | 33,37 |
| **26** | 46 | 2385 | 100 | 32,95 | 32,80 | 32,61 | 35,52 | 31,97 | 33,51 |
| **27** | 9,6 | 425 | 100 | 32,07 | 32,40 | 31,96 | 34,62 | 31,50 | 34,24 |
| **28** | 11,8 | 680 | 100 | 31,65 | 33,74 | 32,41 | 34,37 | 31,42 | 33,34 |
| **29** | 8 | 725 | 100 | 32,01 | 32,80 | 32,52 | 35,33 | 31,28 | 34,14 |
| **30** | 12,5 | 600 | 100 | 36,17 | 37,11 | 36,84 | * | 36,08 | * |
| **31** | 33 | 1750 | 100 | 33,90 | 34,52 | 34,30 | 36,99 | 32,56 | 37,63 |
| **32** | 18,6 | 980 | 100 | 34,58 | 35,23 | 34,78 | 36,55 | 34,07 | * |
| **33** | 20,3 | 985 | 100 | 32,94 | 33,48 | 33,59 | 35,99 | 32,21 | * |
| **34** | 40,8 | 2145 | 100 | 33,12 | 34,60 | 33,87 | 36,96 | 33,71 | * |
| **35** | 9,1 | 460 | 100 | 32,99 | 32,90 | 33,27 | 35,95 | 32,22 | 36,61 |
| **36** | 35,2 | 2365 | 100 | 34,98 | 34,59 | 34,97 | * | 34,64 | * |
| **37** | 13,4 | 1090 | 100 | 32,18 | 32,35 | 32,93 | 36,28 | 31,94 | 35,01 |
| **38** | 36,8 | 2250 | 100 | 33,66 | 34,22 | 34,46 | 35,58 | 32,59 | * |
| **39** | 5,2 | 445 | 100 | 31,85 | 34,26 | 34,36 | 36,64 | 32,55 | 38,87 |
| **40** | 32,3 | 1510 | 100 | 30,08 | 31,44 | 31,35 | 34,47 | 30,79 | 34,67 |
| **41** | 7,9 | 490 | 100 | 30,47 | 30,76 | 29,82 | 32,85 | 29,68 | 32,48 |
| **42** | 61,2 | 4740 | 100 | 33,72 | 32,93 | 31,90 | 33,69 | 31,04 | 30,59 |
| **43** | 4,9 | 385 | 100 | 33,27 | 31,71 | 32,70 | 36,58 | 33,00 | 32,99 |
| **44** | 35,4 | 1305 | 100 | 32,17 | 32,37 | 32,00 | 34,86 | 30,66 | 35,05 |
| **45** | 75,1 | 4385 | 100 | 30,77 | 31,98 | 33,02 | 35,38 | 31,20 | 34,56 |

| **Samples** | **Weight** | **TRNA** | **IRNA** | **ABL1** | **CASC3** | **CDKN1ß** | **HMBS** | **POLR2A** | **TBP** |
| --- | --- | --- | --- | --- | --- | --- | --- | --- | --- |
| **46** | 8,7 | 745 | 100 | 32,73 | 33,37 | 33,64 | 35,66 | 32,85 | 36,88 |
| **47** | 50 | 3830 | 100 | 31,33 | 32,96 | 32,73 | 35,53 | 30,88 | 35,41 |
| **48** | 8,3 | 545 | 100 | 33,18 | 33,08 | 33,48 | 37,11 | 33,42 | 34,44 |
| **49** | 65,2 | 4520 | 100 | 29,97 | 29,83 | 30,60 | 34,74 | 30,97 | 33,63 |
| **50** | 12,7 | 600 | 100 | 29,76 | 30,85 | 31,38 | 33,29 | 30,68 | 31,98 |
| **51** | 53,9 | 4175 | 100 | 31,80 | 31,75 | 32,42 | 34,98 | 31,73 | 33,78 |
| **52** | 33,3 | 1435 | 100 | 32,52 | 33,23 | 32,95 | 35,94 | 32,91 | 37,03 |
| **NTC** |  |  |  | * | * | * | * | * | * |

* Below the detection limit; NTC: no template control
